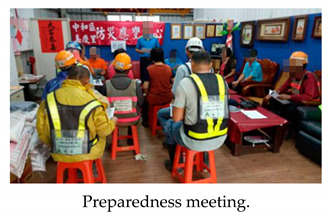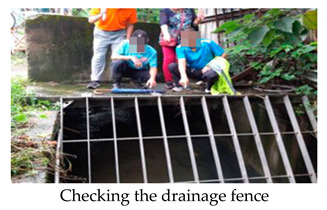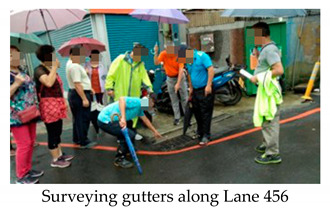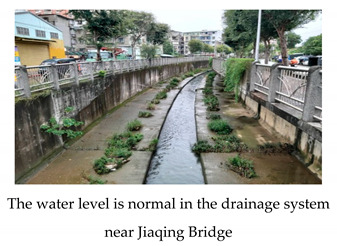# Enhancing Local Disaster Management Network through Developing Resilient Community in New Taipei City, Taiwan

**DOI:** 10.3390/ijerph17155357

**Published:** 2020-07-24

**Authors:** Kai-Yuan Ke, Yong-Jun Lin, Yih-Chi Tan, Tsung-Yi Pan, Li-Li Tai, Ching-An Lee

**Affiliations:** 1 Center for Weather Climate and Disaster Research, National Taiwan University, Taipei 10617, Taiwan; kent0115@gmail.com (K.-Y.K.); yctan@ntu.edu.tw (Y.-C.T.); tsungyi.pan@gmail.com (T.-Y.P.); tailily26@gmail.com (L.-L.T.); 2 Fire Department, New Taipei City Government, New Taipei City 220225, Taiwan; lee.ching.an@gmail.com; 3 Center for General Education, National Taiwan Normal University, Taipei 106209, Taiwan

**Keywords:** resilient community, disaster management, all-hazards approach

## Abstract

Large-scaled disaster events had increasingly occurred worldwide due to global and environmental change. Evidently, disaster response cannot rely merely on the public force. In the golden hour of crisis, not only the individuals should learn to react, protect themselves, and try to help each other, but also the local school, enterprise, non-government organization (NGO), nonprofit organization (NPO), and volunteer groups should collaborate to effectively deal with disaster events. New Taipei City (NTPC), Taiwan, was aware of the need for non-public force response and therefore developed the process of enhancing local disaster management networks through promoting the resilient community since 2009. The concept of a resilient community is to build community-based capacity for mitigation, preparedness, response, and recovery in an all-hazards manner. This study organized the NTPC experience and presented the standard operation procedure (SOP) to promote the resilient community, key obstacles, maintenance mechanism, and the successful formulation of the local disaster management network. The performance of the promotion was evaluated through a questionnaire survey and found that participants affirmed the positive effect of building community capacity through the entire process. In general, the resilient community as the center of the local disaster management work is shown promising to holistically bridge the inner/outer resources and systematically respond to disaster events.

## 1. Introduction

Global warming and environmental changes have led to more frequent and extreme weather events and resulted in disasters of a greater magnitude worldwide. Serious disaster events accompanied by significant casualties repeatedly occurred, such as the 1995 Great Hanshin earthquake in Japan, the 1999 Chi-Chi earthquake in Taiwan, the 2004 Indian Ocean earthquake and tsunami, 2005 Hurricane Katrina in the USA, 2008 Sichuan earthquake in China, 2009 Typhoon Morakot in Taiwan, as well as 2011 Tohoku earthquake and tsunami in Japan. Exposure of persons and assets in all countries has increased faster than vulnerability has decreased, thus generating new risks and a steady rise in disaster-related losses, especially at the local and community level. The impact could be short, medium, and long term and appears in terms of economic, social, health, cultural, and environmental aspects [1].

In the 1995 Great Hanshin earthquake, during the early stage, 34.9% of those in danger survived by themselves, 31.9% escaped with assistance by family members, 28.1% by neighbors/friends, and 2.6% by passerby [2]. Only less than 1.7% of those in need of help were saved by the public force. This investigation indicated that, in such a great-scaled disaster, public force usually could not timely reach all the affected areas. Therefore, the community must be resilient enough to respond by themselves and help each other in the golden hour of crisis events. Community resilience refers to the capacities and capabilities of a human community to “prevent, withstand, or mitigate” any traumatic event [3]. To strengthen community resilience, not only the residents but also neighboring stakeholders, no matter the public sector or private sector, units, or individuals, should join together to form a local disaster management network. It is not easy for the community to organize such a network by itself; hence, the government must invest funding and resources to accomplish this goal. Many studies have shown that to deal with disasters, whether pre-disaster [4,5], in-disaster [6], or post-disaster [7], awareness raising [8] and capacity building [9] are of significant importance, especially at the community level. 

This study aims to present how New Taipei City (NTPC) government, Taiwan, integrated the resources at the local government level and enhance the local disaster management by building a significant amount of resilient community, and begins with why the promotion of resilient community is necessary and how the promotion links to the local disaster management network. The performance is assessed through a questionnaire survey. Two successful cases of community operation are introduced. From the NTPC government’s angle, its experience from nowhere to somewhere is investigated and key obstacles, as well as solutions, are finally identified.

## 2. Materials and Methods

### 2.1. Study Area

**Figure 1:**
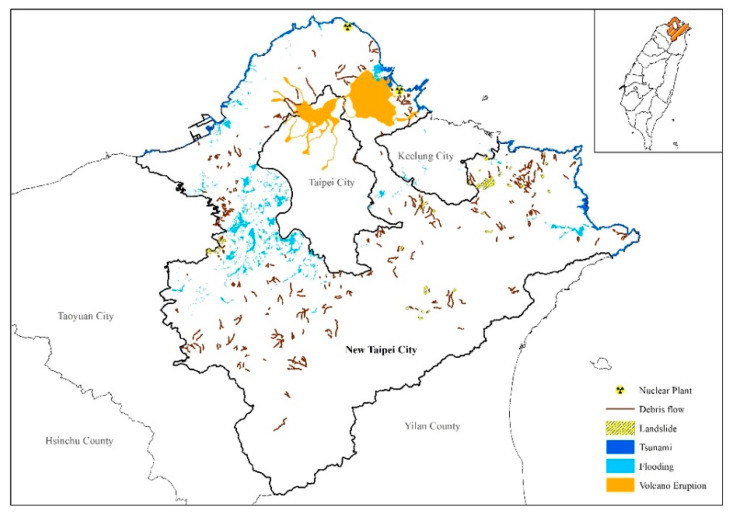
Study Area and Disaster Potential.

New Taipei City, Taiwan, covers an area of 2053 km^2^ with a population of 4 million. There are 29 districts and 1032 villages under NTPC authority. Districts can be categorized into 3 types, i.e., 8 in the urban areas, 15 in the rural areas, and 6 in urban-rural areas. Geologically, NTPC is extremely vulnerable to earthquakes due to the direct pass-through of active Shanchiao fault from the south-west to the north-east. From a topographical perspective, 88% of NTPC is the mountainous area (partly covered by Tatun volcano), and the entire coastline is 126 km long, which means NTPC is prone to geohazards such as debris flows, landslides, volcano eruptions, and tsunamis. Flooding is another disaster event happening frequently due to annual typhoon and torrential rain. Furthermore, two nuclear power plants are situated in NTPC, implying possible nuclear hazards (Figure 1). 

According to the report by the National Fire Agency, Ministry of Interior, Taiwan [10], a total of 42,308 (partly) collapsed buildings, 20,843 casualties, and 87,949 citizens in need of shelter are likely to happen if a large earthquake of scale 6.6 occurred in the center of Taipei Basin. With such kind of catastrophic damage, the public force is unlikely to give support for all affected areas fully and timely. More assistance from private sectors or citizens is necessary, especially those in or nearby the disaster hotspots.

### 2.2. Steps of Promoting Resilient Community 

NTPC’s disaster management system can be divided into three levels, i.e., local government, district office, and community, from the top down. NTPC government was aware of the complex and hazard-prone environment, as well as the abovementioned potential damage which cannot rely on merely the government’s capacity. Therefore, the government thought of enhancing the local disaster management network through matching cooperation between the local units and individuals. To do so, the promotion of the resilient community was considered as the cornerstone. Seven standardized steps were taken to develop a resilient community in NTPC as follows [11].

#### 2.2.1. Step 1. Start-Up Meeting

Stakeholders in the resilient community include the public sector, community residents, and at least one expert in the disaster management field. To coordinate the resilient community promotion, the start-up meeting is hosted. In the meeting, it is vital to make sure the key person in the community, usually the village chief or community committee chairman, understands the benefit of the resilient community and has the willingness to cooperate in the future activities to be hosted. 

#### 2.2.2. Step 2. Activation Workshop

To encourage community participation, it is necessary to arouse public interest through the activation workshop in which the invited expert would give the lecture on the resilient community. Because not all the community had experienced a serious disaster event, the lecture material usually includes not only the concept of the resilient community but also some case studies about disaster scenarios and associated casualties in Taiwan or worldwide. Successful cases of resilient community operation were also delivered to construct the vision and inspire the residents’ participation in future activities. All lecture materials are prepared for the layperson rather than for an expert in order to ensure the lecturer and participants are on the same page.

#### 2.2.3. Step 3. Site Survey and Strategy Development Workshop

**Figure 2:**
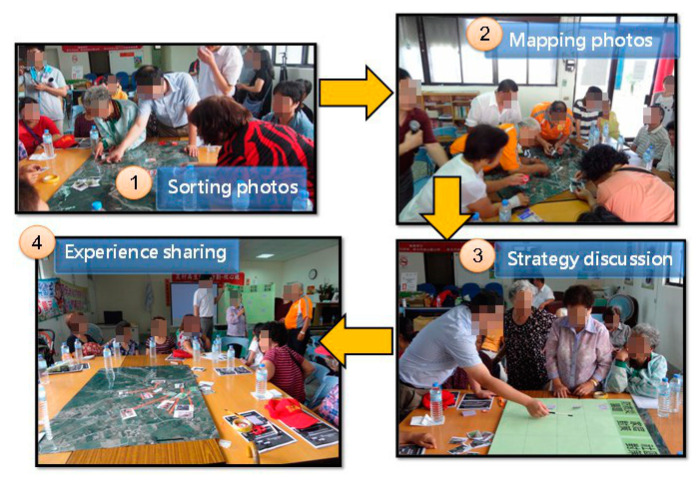
Strategy Development Process.

There has to be a broader and more people-centered preventive approach to disaster risk. Disaster risk reduction practices need to be multi-hazard and multi-sectoral, inclusive, and accessible to be efficient and effective [1]. Therefore, community residents are invited to jointly investigate the environment. Accompanied by experts, residents learn to identify potential/historical disaster hotspots and resources, such as shelter, convenience stores, and public facilities, useful for responding to the disaster event. After the site survey, all participants will furtherly discuss associated strategies through following 4 minor steps (Figure 2):

1. Sorting photo: During the site survey, photos are taken and printed. Participants are asked to sort out the photos into two categories, i.e., disasters hotspot and resource points.
2. Mapping Photo: Those photos sorted in the previous step are pasted on the aero map with stickers near the photo. If the photo is a disaster hotspot, its condition, such as the location and cause/effect of the potential disaster is written down on the stickers; if the photo is a resource point, its function is described.
3. Strategy discussion: With possible disaster conditions and resource points at hand, the expert will help participants discuss strategies to deal with issues from the perspective of the individual, the community, and the local government level. For example, trash sometimes jams the gutter and causes flooding; therefore at the individual level, every resident should be made aware of not dropping trashes in the gutter; at the community level, residents should team up to clean the gutter regularly especially before the flooding season; at the local government level, district office can ask the cleaning contractors to dredge the cutter or provide the community with equipment needed to clean it. Local enterprises and schools can be invited to discuss their role as outer resources to help the community respond to disasters.
4. Experience sharing: The goal of this workshop is to finalize valid strategies mainly by the community; therefore, resident representatives are asked to report the discussed strategies to all the participants and try to reach consensus.

#### 2.2.4. Step 4. Resilient Community Response Team and Action Plan Workshop

##### Community Response Team

**Figure 3:**
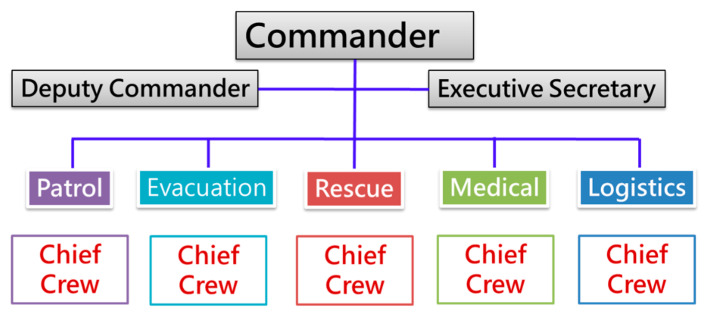
Typical Resilient Community Response Team in New Taipei City (NTPC).

To efficiently carry out strategies in the previous step, the resilient community response team is organized. The typical structure of the response team is shown in Figure 3. It contains five divisions, namely, patrol, evacuation, rescue, medical, and logistics, with their general function as Table 1. The commander, usually the village chief or community committee chairman, supervises the deputy commander and executive secretary, as well as oversees outsourcing and leads the team. The deputy commander supervises the heads of every division and the executive secretary assists the commander and the deputy commander.

##### Community Action Plan

Based on the characteristics of the potential disaster, the community action plan is suggested to include but not limited to the following items.

1. Environmental and disaster risk assessment

The environmental assessment should cover the location of the community, its neighboring geography, social condition, and historical disaster hotspots. The disaster risk assessment must include disaster type the community is facing and associated risk map drawing. The community usually has no capacity of drawing such kind of risk map; therefore, it is advised to utilize some government resources. In Taiwan, the National Science and Technology Center for Disaster Reduction (NCDR) developed the risk map platform (https://dmap.ncdr.nat.gov.tw/) for the public to have access to risk maps of earthquake, landslide, debris flow, flooding, tsunami, and nuclear event nationwide.

2. Community response team and local disaster management network

The community response team is the frontline force to deal with the disaster. According to the experience of all resilient communities promoted by the NTPC government, the general functions of the team were organized as in Table 1. In addition to the community’s strength, outer resources, such as district office, fire department, police department, school, enterprise, volunteers, NGO, and NPO could be invited to formulate a local disaster management network and cooperate pre-disaster, in-disaster, and post-disaster.

3. Resources inventory and management

Community resources mean the equipment such as pump, power generator, fire extinguisher, and power saw owned by the community or facility such as activity center, shelter, and community office managed by the community. However, those existent resources might not fully meet the need in terms of disaster response. The community should periodically update resources inventory and proactively assess the extra demand for resources to deal with the possible disaster. All the resources must have someone be appointed to manage. Some of the duties could be assigned to the community response team member as a suggested division task in Table 1.

4. Sustainable operation mechanism

After the resilient community is established, the top issue is that the community sometimes does not keep on its work due to not having a sustainable operation mechanism to follow. The standard sustainable operation mechanism for the resilient community in NTPC includes the following items:

(1) Regular training: It defines the courses and skill training to behold and its frequency;
(2) Community disaster management database update: It includes the response team member recruitment/retirement, vulnerable residents list update, and equipment maintenance frequency;
(3) Disaster processing record: The community should record the action taken pre-disaster, in-disaster, and post-disaster. It helps review the community action as well as identify defects and weak points of the plan.

The community action plan was discussed and instituted by the residents and the community response team. Stakeholders, such as the school, enterprise, or vulnerable individual/groups in the neighboring area, were welcome to join the discussion. The role of each stakeholder was be identified, e.g., community response team as helpers; residents and vulnerable individuals/groups as help receivers; enterprise as helpers and living material supplier; school as shelter accommodators.

#### 2.2.5. Step 5. Education and Training Workshop

Education and training aim to develop the knowledge and basic skills for community residents responding to disasters and specifically enhance the response team’s capacity to execute their tasks. For the basic knowledge, the courses include disaster response concepts according to the community disaster characteristics. The required skills include basic first aid, such as CPR (cardiopulmonary resuscitation), Heimlich maneuver, and AED (automated external defibrillator) and operation of equipment such as fire extinguishers, pumps, power saws, etc. This course is suggested to be hosted at least once per year.

#### 2.2.6. Step 6. War Game or Drill

The community response team members could practice their tasks and skills through the war game or drill. War game helps test the validity of the action plan established in step 4, and the drill can further test the skills learned from step 5. In NTPC, not only the community response team but also stakeholders in the neighboring area, such as staff from the district office, the local fire department, school staffs, and enterprise partners are role players. Table 2 is the typical scenario designed for an earthquake drill in NTPC. A few key principles are suggested as follows:

1. Scenarios must correspond to community characteristics in terms of single disaster or complex disaster.
2. Self-protection skills of individuals could be exercised, such as “Drop”, “Cover”, “Hold On” during the earthquake.
3. The disaster scale should be designed properly so that the community must and could react. If the scale is too small, then no significant damage will highlight the necessity for community response; if the scale is too large, most community members might lose their capability due to casualties resulting in malfunction of the team.
4. Every division in the community response team should have the chance to familiarize themselves with their tasks and required skills.
5. Coordination and communication among the response team, stakeholders, and public/private agencies should be tested.
6. The community should understand the evacuation routes to the shelter as well as arrange and test the transportation for evacuation.
7. Collaboration between the district office and the community team to open the shelter should be exercised.

#### 2.2.7. Step 7. Exhibition of Resilient Community

Upon completion of resilient community development, posters and videos are made showing the annual activities and joint efforts achieved by the community, government, school, and enterprise. The community response team member share experiences with those from other communities/villages who have never joined the resilient community workshop. The purpose is to not only encourage the ongoing involvement in this developed resilient community but also inspire other villages’ participation shortly.

### 2.3. Questionnaire Survey 

To evaluate the effect and performance of promoting a resilient community, an anonymous physical questionnaire survey was conducted after we finished each resilient community for that year. The participants were informed that participation was voluntary and the participants’ willingness to return the completed questionnaire indicated their consent to participate in this study. Eight key questions were asked as follows:

Q1: Do you understand the disaster risk of your community after the workshop?

Q2: Do you feel developing a resilient community and building capacity is necessary?

Q3: Has your community built a feasible action plan after the workshop?

Q4: Do you understand the tasks of the response team?

Q5: Are you willing to become a member of the response team?

Q6: Have you learned basic medical skills and been capable of performing it when necessary?

Q7: Have you learned the fire-fighting skills and been capable of performing it when necessary?

Q8: Is retraining necessary for the community?

Q1 and Q2 checked if the participants were aware of the disaster risk and management; Q3 checked if the community action plan was built and valid; Q4 and Q5 checked if the participants understood the tasks they should perform while they became response team members; Q6 and Q7 checked if basic skills were well taught; Q8 checked the necessity of hosting retraining courses, and is linked to the maintenance mechanism in Section 4.2.

Despite the eight key questions, only age and gender information were collected; therefore, no personal information of any specific individual could be exposed.

## 3. Results

### 3.1. Performance Survey

**Figure 4:**
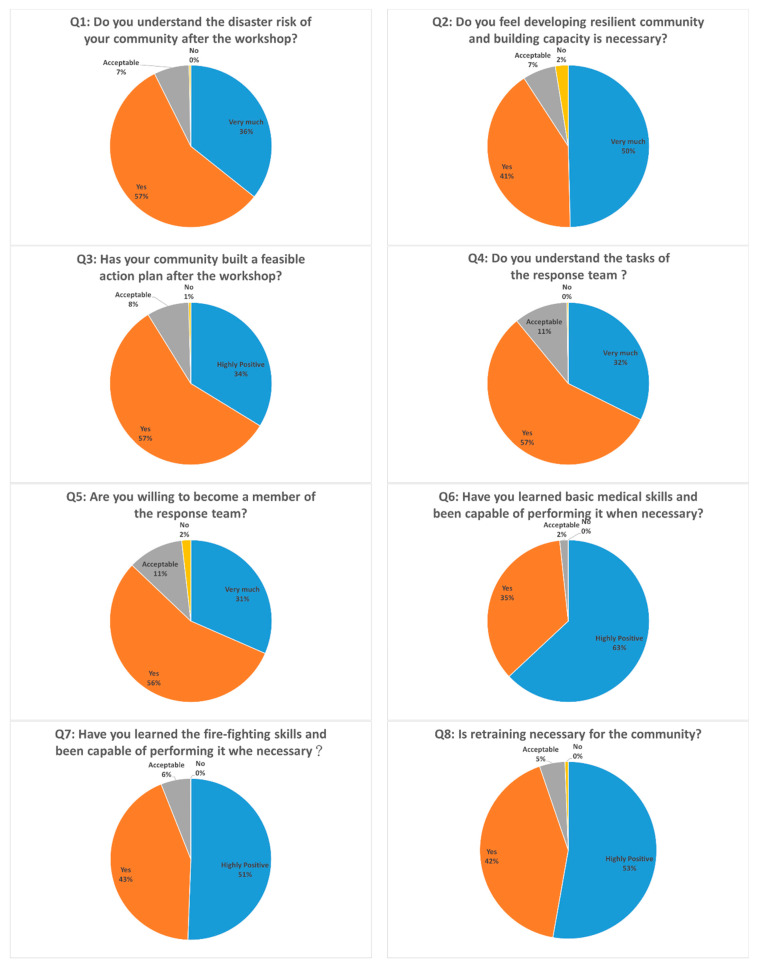
Results of the Questionnaire Survey.

Table 3 shows the age distribution of respondents who joined the workshops hosted by the NTPC government in 2019. We kindly asked every participant to do the questionnaire for us right after the workshop; therefore, the response rate was 100%. From a total of 1180 participants, including 520 males and 660 females from 33 communities, more than 80% of them were over 50 years old, and more than 50% were over 60 years old. The aging population phenomenon is very common in rural areas of NTPC which are usually prone to high disaster risks. It implies that their mobility to react to disaster events is relatively low before the promotion of a resilient community. The questionnaire was designed to confirm the contribution of promotion, and results are shown in Figure 4.

The survey has shown that, after 7-steps of promotion as described in Section 2.2, 93% of the participants realize the risks they are facing and 91% agree with the necessity to develop a resilient community; 91% believe that the action plan we helped them build is feasible; 89% understand the tasks of the response team and 87% are willing to serve the community as a team member; 98% and 94% think that they had well learned and were ready to perform basic medical skills and fire-fighting, respectively; 95% also thinks retraining is important for the community. Overall, about 90% of the participants’ awareness was raised and the capacity to deal with community-based disaster events was established. It indicates the triumph of resilient community promotion and implies its contribution to the successful community operation introduced in the next section. 

### 3.2. Successful Cases of Community Operation

Two case studies are introduced to demonstrate how the established resilient community reacts pre-disaster, in-disaster, and post-disaster. Those cases may not have been catastrophic events but showed how the community spontaneously mobilized after the training received through building community resilience.

#### 3.2.1. Jiaqing Village—Preparedness before the Typhoon Event

**Figure 5:**
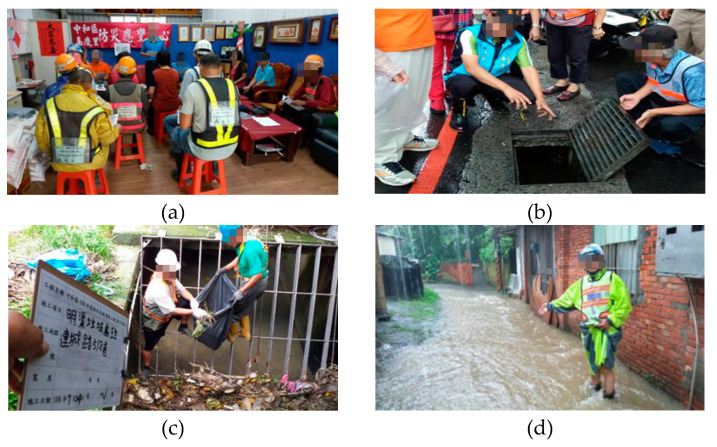
(**a**) Preparedness meeting; (**b**) patrolling the drainage system; (**c**) cleaning the drainage system with district cleaning contractor; (**d**) identifying and recording the flooding situation for future improvement.

Jiaqing Village, an urban village located in Zhonghe District, is the resilient community that started in 2019. This village was prone to flooding, earthquake, and fire. After the village was trained and the community response team was organized, it progressively operates whenever there is a typhoon coming (Figure 5). The village chief, as the response team commander will host a preparedness meeting and assign tasks for the team. The biggest concern is to prevent the low-lying area from flooding; therefore, team members were sent to the gutter and drainage outlet where garbage is easily accumulated. Once waste was found stuck in the drainage system, the team notified the district cleaning contractor and cleaned the site together. Occasionally, if the cleaning of the drainage system could not prevent the flooding from happening, the team recorded the situation for the village chief to discuss improvement measures thereafter. 

#### 3.2.2. Baiyun Village—Responding to a Local Landslide Event

Baiyun Village, a mountainous village located in Xizhi District, is a resilient community stated in 2016. After six months of solid training and immediately after the community drill was performed on 8 October 2016, a landslide event occurred due to Typhoon Aere in the early morning of 9 October. The Village chief, Jun-di Chen, immediately assembled the community response team as well as reported the situation to the Xizhi District Office and NTPC Fire Department as soon as he was notified by the residents who spotted the event. Eight team members were called in and approached the disaster site to evacuate people by knocking on doors one after another. Once the government forces arrived and took over the frontline, the community response team helped set up the cordon to prevent residents from entering the disaster site. The team also helped the public force establish the command post in the nearby area to monitor disaster development and timely response. Finally, when the situation was under control, the response team moved to the shelters and took care of the residents who had evacuated earlier. In total, 34 people took shelter in the Baiyun Activity Center with no casualties reported.

## 4. Discussion

### 4.1. Obstacles and Solutions

The resilient community developed in NTPC has by far been running for three phases as follows. Most problems were identified in phase 1 and solutions were given accordingly in phases 2 and 3. 

#### 4.1.1. Phase 1: Resilient Community 1.0 (2009–2015)

NTPC has launched the resilient community since 2009. Until 2015, only 13 resilient communities were developed by a few NTPC departments. The speed of promotion is quite slow because the NPTC government was unfamiliar with the concept of the resilient community and need help from certain universities who have associated expertise and enough manpower to host the workshops and activities described in Section 2.2. 

During the first phase, key factors impeding the promotion were identified as follows:

##### Insufficient Willingness

In general, residents usually lack the willingness to participate in the resilient community workshop from the beginning due to three reasons. First of all, they think that if no serious disaster happened before then why would there be one in the future. Next, there is already some structural protection in the community such as the dike or pumping stations/machines to prevent flooding and the retaining wall to prevent from hillslope disaster. They feel quite safe with those protection measures. Finally, even if a disaster indeed happened, the government would come and help because the government must save the citizens. 

##### Environmental Variety

There are varying conditions in different communities. The community is usually prone to hillside disaster and debris flow in the rural area especially in the mountainous area; prone to earthquake and fire in the urban area especially with densely distributed old buildings; and prone to flooding in the low-lying area. Therefore, there is no “one size fits all” approach for community resilience building [12].

##### Time-Consuming and Financial Concern

Although the goal of the resilient community is building capacity for it, the NTPC government specifically asks the public sector such as district office and local fire department corps and branch to progressively join associated activities. Therefore, a great amount of time and involvement from the community and public sectors is required. It usually takes a minimum of 3–6 months to develop a base-type resilient community and up to 2 years to finish the complete-type resilient community. The minimum requirement for a base-type resilient community is to raise the residents’ awareness and train their basic skills. For the complete-type resilient community, the 7 steps in Section 2.2 should be strictly followed and their performance tracked to ensure a fully built capacity. It would cost 10,000 to 13,000 USD to hire the expert/team to finish one complete-type resilient community. There are 1032 villages in NTPC, and the total expense would exceed 10 million USD for all.

##### Various Authority Concerned 

The different authorities concerned are entitled to deal with different disaster types. For example, in NTPC, the Water Resources Department and the Agriculture Department promote resilient communities prone to flooding and debris flow, respectively. It is not be a problem if the community has only a single disaster type. However, it is very common that the community has more than one disaster potential. More than one department can invest in the same community if they wanted to, resulting in the duplicate investment and waste of government resources, furthermore, harming the government’s general interest. One other issue is that every department in the local government is a subordinate agency of certain authority in the central government which institute the policy to promote the resilient community. For example, the Soil and Water Conservation Bureau (SWCB) under the Council of Agriculture supervises the Agriculture Department in NTPC. They focus only on debris flow and train the residents accordingly. On the other hand, the Water Resources Agency supervises the Water Resources Department in NTPC to build flood-proof capacity for the community. As a result, not all communities receive the same training and build the all-hazards response code.

The abovementioned four obstacles account for the “Integrated Resilient Community Program” launched by the NTPC government in phase 2 and the necessity of establishing a maintenance mechanism, as shown in the following section.

#### 4.1.2. Phase 2: Resilient Community 2.0 (2016–2017)

**Figure 6:**
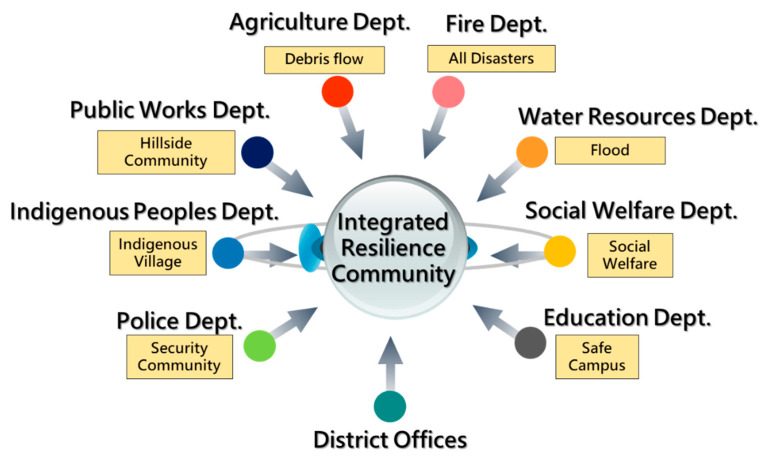
The Concept of Integrated Resilience Community with Associated Authorities Concerned in NTPC.

From 2016, NTPC launched the “Integrated Resilient Community Program” to assemble all resources, including funding and manpower, from eight departments (Water Resources Bureau and the departments of Fire, Agriculture, Social Welfare, Education, Public Works, Police, and Indigenous Peoples) (Figure 6). It ensures not only the optimal utilization of the local government’s resources but also the consistent procedures for all departments to follow and promote resilient communities.

In phase 2, the school played quite an important role in the local disaster management network. The Ministry of Education had initiated the campus safety program in 2003, and the focus was on building school internal capacity until 2010. After 2011, schools were asked to gradually cooperate with nearby villages and communities in the context of disaster management. School and district activity centers are two major facilities in Taiwan to shelter the refugees in a disaster event. The community and school must work together while opening the shelter. Besides, both of them could collaborate in medical service, mental caring, patrolling disaster hotspots, and dealing with small-scaled disaster events if needed. Such cooperation is practically valid because most students, even teachers, are from a neighboring community and therefore a tight bonding already exists. The only movement needed to enhance the link and push forward is asking both parties to attend the resilient community workshop and discuss the terms of cooperation in the context of the local disaster management network. Schools, especially at the university level, can also help build resilience capacity for the community [13].

#### 4.1.3. Phase 3: Resilient Community 3.0 (2018~)

In phases 1 and 2, all of the resilient communities were promoted by the local government’s departments with help from certain universities. However, building community capacity to deal with disasters is the legal duty of the district office in Taiwan. To help the district office learn and promote the resilient community by itself, the community consultant team was organized by the NTPC government in 2018. It hires experts specialized in community disaster management to train the district offices to promote the resilient community through the seven-step process. 

Besides, the resources from enterprises were specifically introduced to the community in phase 3. As is well known, the key to successful enterprise disaster management is the development of business continuity planning (BCP). However, BCP functions more internally than externally. It means, with BCP, the enterprise knows how to deal with disaster by itself whether in terms of mitigation, preparedness, response, or recovery. What the NTPC government tries to achieve is to develop a cohesive local disaster management network that involves the collaboration of community, public sector, schools, and enterprises. The enterprise is the last piece to complete such a network. Not all enterprises are suitable to join the network. The enterprise must meet three NTPC criteria such as positive image, enough scale, and high willingness. The NTPC government or district office will sign the MOU with the enterprise after it is chosen. To build tighter bonding among stakeholders, the enterprise is invited to join the resilient community activity and discuss cooperation or action plan as mentioned in Section 2.2.4. Other than direct financial support to the community or public sector, there are various ways in which the enterprise can play a role in the local disaster management network. For example, the Mitsui Outlet Park in Linkou joined the drill hosted by the Linkou District Office and provided hot meals and medicines for nearby communities; Yulon Group, well known for its Yulon Motor Co., Ltd. offered Xindian District Office vehicles to evacuate community refugees. Through helping the local government and community, the enterprise can not only fulfill corporate social responsibility (CSR) but also enhance its image from the public sector’s media propaganda.

### 4.2. Suggested Maintenance Mechanism of Resilient Community

The maintenance of the resilient community is usually harder than its development; therefore, it is suggested to employed four measures as the NTPC government did and keep the heat on.

#### 4.2.1. Retraining Courses

Retraining is vital as shown by the questionnaire survey (Q8). Various courses could be chosen from the following depending on the community’s needs.

1. Tasks review of the community response team: New members will join the community response team now and then. It is of great importance to make sure each member, whether senior or newcomer, knows his/her task well.
2. Collection and reporting of disaster information: With the popularization of smartphones, more apps are available for collecting disaster information and uploading it to the cloud platform. The community should learn which technical tool is more suitable to the community and how it functions. All disaster information collected could be reserved in a community database for future review.
3. Advanced disaster response skills: Basic skills such as CPR, Heimlich maneuver, and fire extinguisher operation were taught while developing a resilient community. Advanced skills, such as patient moving, escape from the fire scene, and responding with the tool at hand (e.g., making slippers with old newspapers; making simple toilets with paper box and plastic bag) are suggested in the retraining courses. Considering the COVID-19 pandemic in 2020, epidemic prevention is also suggested to be included in the retraining. Thereby, every trainee could be a community watcher and help spread epidemic prevention knowledge and support the government’s action if necessary. The selection of skills is not limited to specific disaster types that the community is most likely to confront. The advanced skill training aims to make the community function in an all-hazards response manner.
4. War game: Every disaster management action plan should be periodically reviewed and tested. At the community level, war game is a less costing and less time-consuming way to validate the plan compared to drill. However, the design of a proper war game is still not easy for the community. They should deeply consider the potential risk and transform it into disaster scenarios for strategy discussion. They will also have to manage inner resources and seek additional outer resources. Usually, inviting experts or public sector personnel to join the war game would help the community deliver more insightful outcomes.

#### 4.2.2. Equipment Subsidization

Skills training and raising awareness are compulsory for community residents to increase their chance of survival in the catastrophic disaster event. With the right tools and equipment, the core function of self-help and mutual help could be even more effective. The NTPC government supports certain funding for the community to purchase equipment upon the completion of a resilient community establishment. The community could buy the equipment according to a predefined list which includes evacuation bag, disaster prevention hood, helmet, first-aid kit, stretcher, walkie talkie, pumps, fire extinguisher, trolley, power saw, power generator, emergency ration, etc. The purchased equipment should be listed in the community action plan and be maintained regularly. The response team member must be trained to operate it. 

#### 4.2.3. NTPC Resilient Community Certificates

**Figure 7:**
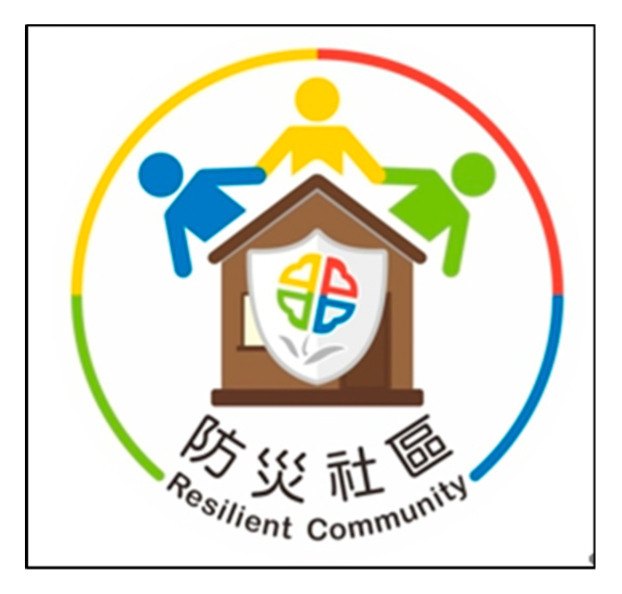
NTPC Resilient Community Certificate.

Issuing the resilient community certificate to those progressively engaged in associated activities and who made solid achievements would raise the community’s sense of honor and make it more likely to keep on the operation. NTPC government initiated the certificate application program in 2018. The community receives the NTPC certificate (Figure 7), and it proves the following criteria have been met:

1. Environmental risk assessment: The identification of disaster potential and associated strategies must be delivered.
2. Community disaster management database: Including the identification of vulnerable people in the community, inventory of equipment, list of community residents with special skills and who can help respond to disaster, and contact list of outer resources such as police department, fire department, volunteer, school and enterprise.
3. Community response team: Including the head and crew of the five-response team divisions. It is batter if the enterprise and school can join as a support division.
4. Skill training: Including basic skills introduced in Section 2.2.5.
5. Drill: Including the script with properly designed disaster scenarios and the actual role-playing of 5 team divisions.

#### 4.2.4. Tracking Community Performance

Ideally, after the resilient community is established, it should consistently and spontaneously operate by itself; nevertheless, this is usually not the case in reality. Without the government’s supervision or expert’s assistance, some communities fail to keep on with the work. To avoid it, the NTPC government designed a simple performance tracking table (Table 4) and asks the community to fill it in whenever a disaster happens or is expected to come. 

The Table is separated into 5 operation types, valid not only for operation during the disaster event but also for mitigation measures on normal days. The following are some suggested actions that the community can take.

1. Mitigation: Including routine education, skill training, drill/war game, environment patrol, disaster information;
2. Preparedness: Including hosting preparedness meeting, equipment inventory, real-time weather monitoring and early warning, checking vulnerable people’s condition and need, patrolling areas prone to disasters, and shelter opening preparedness;
3. Report in: Once the disaster is spotted, reporting to the community and the authority concerned for timely response, as well as to associated private sectors such as water company or power company for assistance;
4. Response: Including dealing with disasters such as removing fallen trees, fire-fighting, identifying risk area and setting up cordon; evacuating people in the high-risk area; helping public sectors such as opening shelter, traffic control, and setting up command post; taking care of wounded by first-aid, caring for, and moving patients;
5. Recovery: Including environment cleaning, recovery, and rebuilding.

## 5. Limitation and Challenges

### 5.1. Aging Population.

As shown in Table 3, the population is aging in NTPC rural areas. Young people leave their hometown to seek more work opportunities, which leave the elders more vulnerable to disasters. The aging population is not a unique problem to Taiwan. Many developed countries, such as Japan, Italy, Finland, Portugal, and Greece, have this kind of social problem. Responding to disaster requires mobile manpower to execute the tasks as designated in Table 1. To have more young people engage in community-based disaster management, the government should help improve the employment market in rural areas to attract young residents’ return or stay. It also implies the inseparability of disaster-related and social-economic issues in the era of public engagement in disaster management.

### 5.2. Lack of Real Experience

It takes a disaster to learn a lesson. However, most people never really suffer from a medium to large scale disaster, not to mention a catastrophic one. What is taught in the resilient community workshop is the concept of self-help and mutual-help, as well as basic response skills. We never know if the residents could apply the concepts and skills perfectly during a disaster event. Therefore, the retraining courses should be hosted persistently. Moreover, most of the public lacks the experience of dealing with post-disaster recovery. It is time for the community to participate in pre-disaster recovery planning with the government to envision the potential damage and associated recovery work. 

### 5.3. Insufficient Funding Support

After the entire training of a resilient community, most residents recognize its necessity and are willing to continue running it. The only problem is where the funding support comes from for consistent operation. Although the NTPC government offers the community certain equipment, it is usually not enough regarding the regular operation, emergency response, and administrative works. More funding contributions from public and private sectors shall be needed. The government should put more effort into matchmaking between the needs of the communities and the resources from enterprises.

## 6. Conclusions

Extracting from NTPC experience, this research has proposed the SOP to promote the resilient community, identified the key obstacles, suggested the maintenance mechanism, and shown the successful formulation of the local disaster management network. The policy to deal with disaster in NTPC is the “top-down” guidance with “bottom-up” implementation. In this manner, responsibilities and initiatives could be well balanced between residents and the government [14]. The network involves the community, local government, district office, school, and enterprise. Those network members are invited to join the workshops and associated training for collaborative learning and developing a viable joint action plan. Therefore, it is expected that, during a major incident or disaster (MID), the resilient community, school and enterprise could all play a role when the local government requires flexible surge capacity (FSC). Surge capacity (SC) means the ability to increase staff, stuff, structure, and system (4S) rapidly and effectively in the affected areas. FSC indicates the capability to scale up and down resources in a fast, smooth, and productive way [15]. The community could provide manpower to help local government in many ways such as, but not limited to, evacuating vulnerable people, opening shelters, managing living supplies/materials, and identifying disaster hotspots. With that assistance, the government could focus more on addressing hardest-hit areas and situations. Since this study shows a promising non-structural method to enhance the local disaster management network, any country or government willing to intensify the capacity of disaster management at the community level could follow NTPC’s steps and avoid the obstacles.

## Figures and Tables

**Table 1:** The Function of the Community Response Team in NTPC.

Team Division	Function	
	Pre-Disaster	In-Disaster and Post-Disaster
Patrol	Understanding and periodically patrolling the disaster potential area and hotspot. Eliminating disaster factors in advance, such as cleaning gutters.	Monitoring weather and patrolling disaster potential area. If a disaster condition is spotted, send messages to the community command center and make records. Setting up a cordon around a disaster point and prevent from a passerby in.
Evacuation	Tabulating and periodically updating the vulnerable residents, such as elderly, incapable people and those living in disaster potential areas. Planning evacuation route. Making and periodically updating the evacuation map.	Reminding and assisting the residents, especially the vulnerable residents, to evacuate in an emergency. Making sure the evacuation route is safe and not blocked. Helping traffic control in vital traffic intersection and direct the evacuating people.
Rescue	Maintaining existing equipment and assess the need for additional equipment based on disaster type and potential in the community. Being familiar with the equipment operation through periodically training.	Keeping smooth telecommunication by preparing walkie-talkie. Preparing the equipment and applying it in a small-scaled disaster event, such as putting out a small fire with a fire extinguisher or sawing a fallen tree into pieces and removing it to avoid traffic congestion. If residents were trapped due to serious events, trying to identify their location and asking support from the authority concerned.
Medical	Being proficient in first aid and caring skills Periodically training residents with those medical skills. Preparing items for medical purposes, such as first-aid kit and stretcher.	Helping injuries in need of first aid. Guide outside medical resources to people in need. Helping local governments open shelters and prepare living supplies. Mentally comforting the refugees scared by disasters.
Logistics	Assessing the living material, such as drinking water, food, and medical needs, required during a disaster event. Tabulating and periodically updating the community response team members. Helping the local government maintain shelters.	Helping local governments open shelters and prepare living supplies. Helping refugees register when they arrive at the shelters and distributing living supplies. Supporting the other four response team divisions.

**Table 2:** Typical Scenarios Designed for Earthquake Drill in NTPC.

Scenario	Situation
Scenario 1	Self-protection, such as “Drop”, “Cover”, “Hold On” exercise at the time of an earthquake.
Scenario 2	Community response team mobilization and preparedness.
Scenario 3	Preparedness for opening shelter by logistic division.
Scenario 4	The assistance of refugee evacuation to the shelter by evacuation division.
Scenario 5	Patrol division surveys the area and calls for help from the rescue division upon locating damage.
Scenario 6	Assistance by logistics division in shelter opening, such as registration, food sharing, and related operations. Living supply may come from the enterprise.
Scenario 7	First-aiding the physically wounded people or caring for the traumatized people by medical division.
Scenario 8	Rescue division puts out small-scaled fire induced by the earthquake
Scenario 9	Recovering the environment by the entire response team and community residents.

**Table 3:** The age distribution of questionnaire respondents.

Age	~20	21~30	31~40	41~50	51~60	61~70	71~80	81~
Number of Participants	13	23	48	126	358	441	143	28
Percentage	1%	2%	4%	11%	30%	37%	12%	2%

**Table 4:** Resilience Community Performance Tracking Table (Example).

Event: Typhoon Mitag				
Operation Duration			Location(s)	Operation Type
	Date	Time	Lane 518, Liancheng Rd. Gutter along Lane 456, Liancheng Rd. Drainage system near Jiaqing Bridge MRT construction site	□Mitigation ■Preparedness □Report in □Response □Recovery
From	2019.09.30	10:00 a.m.		
To	2019.09.30	11:17 a.m.		
Operation Process Note				
Central Weather Bureau issued the land waring of Typhoon Mitag at 20:30, September 29th, 2019. Commander Wu hosted the preparedness meeting Center at 10:00, 30 September 2019, and assemble the leader and crew of 5 divisions of the response team as well as the Taipei Mass Rapid Transit (MRT) construction site manager. Garbage accumulated at the drainage fence in Lane 518, Liancheng Rd. was reported to the district office, and removed by the cleaning contractor. No garbage spotted in the gutter along Lane 456, Liancheng Rd. The water level is normal in the drainage system near Jiaqing Bridge. No flooding in the MRT construction site.